# Biomarker selection and a prospective metabolite-based machine learning diagnostic for lyme disease

**DOI:** 10.1038/s41598-022-05451-0

**Published:** 2022-01-27

**Authors:** Eric R. Kehoe, Bryna L. Fitzgerald, Barbara Graham, M. Nurul Islam, Kartikay Sharma, Gary P. Wormser, John T. Belisle, Michael J. Kirby

**Affiliations:** 1 Department of Computer Science, Colorado State University, Fort Collins, CO 80523 USA; 2 Department of Mathematics, Colorado State University, Fort Collins, CO 80523 USA; 3 Department of Microbiology, Immunology & Pathology, Colorado State University, Fort Collins, CO 80523 USA; 4 Department of Medicine, New York Medical College, Valhalla, NY 10595 USA

**Keywords:** Infectious diseases, Mathematics and computing, Metabolomics

## Abstract

We provide a pipeline for data preprocessing, biomarker selection, and classification of liquid chromatography–mass spectrometry (LCMS) serum samples to generate a prospective diagnostic test for Lyme disease. We utilize tools of machine learning (ML), e.g., sparse support vector machines (SSVM), iterative feature removal (IFR), and *k*-fold feature ranking to select several biomarkers and build a discriminant model for Lyme disease. We report a 98.13% test balanced success rate (BSR) of our model based on a sequestered test set of LCMS serum samples. The methodology employed is general and can be readily adapted to other LCMS, or metabolomics, data sets.

## Introduction

Early Lyme disease develops days to weeks following the transmission of *Borrelia burgdorferi* to a human host via an *Ixodes* tick. Typically a patient will develop an *erythema migrans* (EM) skin lesion and non-specific symptoms including fatigue, malaise, and joint and muscle pains^1^. Although an EM skin lesion is the most common manifestation of Lyme disease and is used for clinical diagnosis in endemic areas, not all patients develop or notice an EM skin lesion^1,2^. Additionally, *southern tick-associated rash illness* (STARI) also causes a characteristic EM-like rash and the geographic expansion of its associated vector *Amblyomma americanum* into Lyme disease endemic areas makes it difficult to accurately diagnose Lyme disease solely by the presence of the characteristic skin lesion^3,4^. The diagnosis of early Lyme disease is further confounded by the reliance of current diagnostics on a serological response that might not be fully developed early in infection and is not able to distinguish between current and past infection^2^. These pitfalls in the current Lyme disease diagnostics invite assessment of non-immune reliant diagnostic approaches.

Previously, we provided proof-of-concept studies for the use of metabolomics to identify host metabolic profiles that could be used as a diagnostic marker of early Lyme disease^5,6^. The classification tools developed were based largely on least absolute shrinkage and selection operator (LASSO) statistical modeling that worked well when the liquid chromatography–mass spectrometry (LCMS)) data of the training and tests sets were collected at the same time (i.e. during the same instrument run)^7^. However, we subsequently realized that the test accuracy faltered when a temporal difference existed for the collection of training and test sample data. This batch effect was in part hypothesized to be due to the sparsity parameters used for LASSO feature selection and in the normalization and imputation approaches used.

In this paper, we use sparse support vector machines (SSVM), a machine learning (ML) tool, to select an optimal set of metabolic biomarkers and then build a metabolite-based diagnostic for Lyme disease^8^. We begin with the hypothesis that feature vectors, or the vectors of metabolite peak areas, for patients with Lyme disease and their healthy counterparts are separated in space when restricted to some reduced set of discriminatory biomarkers. This is the base assumption of sparse, or minimal feature, models for feature selection. Uni-variate statistical tests, e.g. t-tests, identify individual biomarkers that may separate the data^9–11^. In contrast, the multi-variate methods employed here select sets of biomarkers that discriminate as a group by exploiting higher dimensional separation between different metabolic classes. Multivariate models in statistics and ML, such as partial least squares-discriminant analysis (PLS-DA), kernel support vector machines, deep learning networks, and decision trees, can over-fit when training on data sets with many features and relatively few samples^12–14^. This may be mitigated through hyperparameter tuning: controlling the balance between training and validation accuracy in a cross-validation experiment. Using a sparsity inducing penalty in the SSVM optimization problem reduces the number of parameters available to the model and serves to prevent over-fitting by regularizing the high-dimensional model.

ML for classification tasks in metabolomics has seen success for more than a decade. support vector machines (SVM), along with other ML models, have been applied on nuclear magnetic resonance (NMR), LCMS, and gas chromatography–mass spectrometry (GC–MS) metabolomics data, yielding high accuracy and low feature count models for potential metabolite-based diagnostics for conditions such stress, pneumonia, and cancer^15–19^. Evaluations of several ML methods across many different types of metabolomics data can be found^19,20^. In particular, SSVM and support vector machines with recursive feature elimination (SVM-RFE) have been successful in identifying important metabolic biomarkers for different cancers^17,18^. A review of the various predictive and ML models that have been used in metabolomics data can be found in Ghosh et al.^21^.

Previously, sparse linear statistical models, such as LASSO and elastic net, have been used to identify serum metabolite biomarkers and build classification models for distinguishing specific Lyme disease manifestations from healthy controls^5,6,22^. Using SSVM with iterative feature removal (IFR), we improve upon these previous methods, and show that our selected biomarkers and classification model yields greater than a $95\%$ balanced succes rate (BSR) on a sequestered (held-out) test set of serum samples; potentially paving the way for a metabolite based diagnostic test for Lyme disease^23^.

## Results

### Method overview

Early Lyme disease and healthy control serum samples, previously analyzed by LCMS as two separate batches (discovery/training and test), were utilized in this study^24^. A total of 118 training and 118 test serum samples were included. The LCMS data acquired previously were processed using XCMS^24,25^. A list of 4851 features were detected in the training samples. After the untargeted selection in XCMS we checked for missingness in the data to identify features with missing values in more than 80% of training samples (both the Lyme disease and the healthy groups)—none of features met this criterion. The abundance value for each feature was transformed by either the $\log$ transform, standardization (mean $=0$, variance $=1$), median-fold change normalization, or left untransformed^26^. Missing data were imputed using the *k*-nearest neighbors (KNN) algorithm. Uniform manifold approximation and projection (UMAP) was applied as a visualization tool for identifying possible batch effects in the data^27^. To bring together sample-batches of the same group, we utilized an IFR algorithm, Algorithm 1, paired with a SSVM classifier to identify and remove batch-discriminatory features^8,23^. This was performed for the data generated by each transformation scheme.

Once sample-batch effect features were removed, feature selection for differentiation of Lyme disease vs healthy controls was performed with *k*-fold feature selection (*k*FFS), using SSVM as the classifier. We obtained a selected feature set for each data transformation scheme and these features were then combined, and the raw LCMS and LCMS/MS data of each selected feature evaluated to determine appropriateness as a potential classifying feature (i.e. mono-isotopic vs isotopic ion, intact vs insource fragment ion, and ion intensity). This resulted in a final biosignature of 42 high quality features. These were targeted in both the training and test samples’ data in the Skyline software to ensure accurate peak picking^28^. As a final step abundance data acquired via Skyline were *log* transformed, used to train an SSVM classifier with training samples’ data, and tested against the test samples’ data. The pipeline described is provided as python scripts contained in our github repository^29^—the repository contains all python libaries, scripts, and data necessary to reproduce the results of the paper. However, due to the random choice of partitions in the cross-validation scheme used in both IFR and *k*FFS small differences in the resulting feature sets may occur.

### Evaluation of transformation and imputation methods

Prior to the development of a differentiating biosignature and classifier, we evaluated 18 different combinations of transformation and imputation methods with the 4851 features found in training samples. This included median imputation, knn imputation, half-minimum imputation, standardization, log transformation, quantile normalization, and median-fold change normalization^26,30^. This demonstrated that KNN imputation with log transformation on training samples provided the highest mean fivefold cross-validation accuracy (99.8%, 0.3%) when an SSVM classifier was applied. Median imputation with log transformation performed similarly (99.7%, 0.4%). Both standardization and median-fold change normalization obtained relatively high accuracy scores with low standard deviations when paired with KNN imputation. Thus, four transformation-imputation methods were moved forward for biosignature development. The complete results of this experiment can be found in the Supplementary_Data directory of our github repository^29^.

### Batch correction

**Figure 1:**
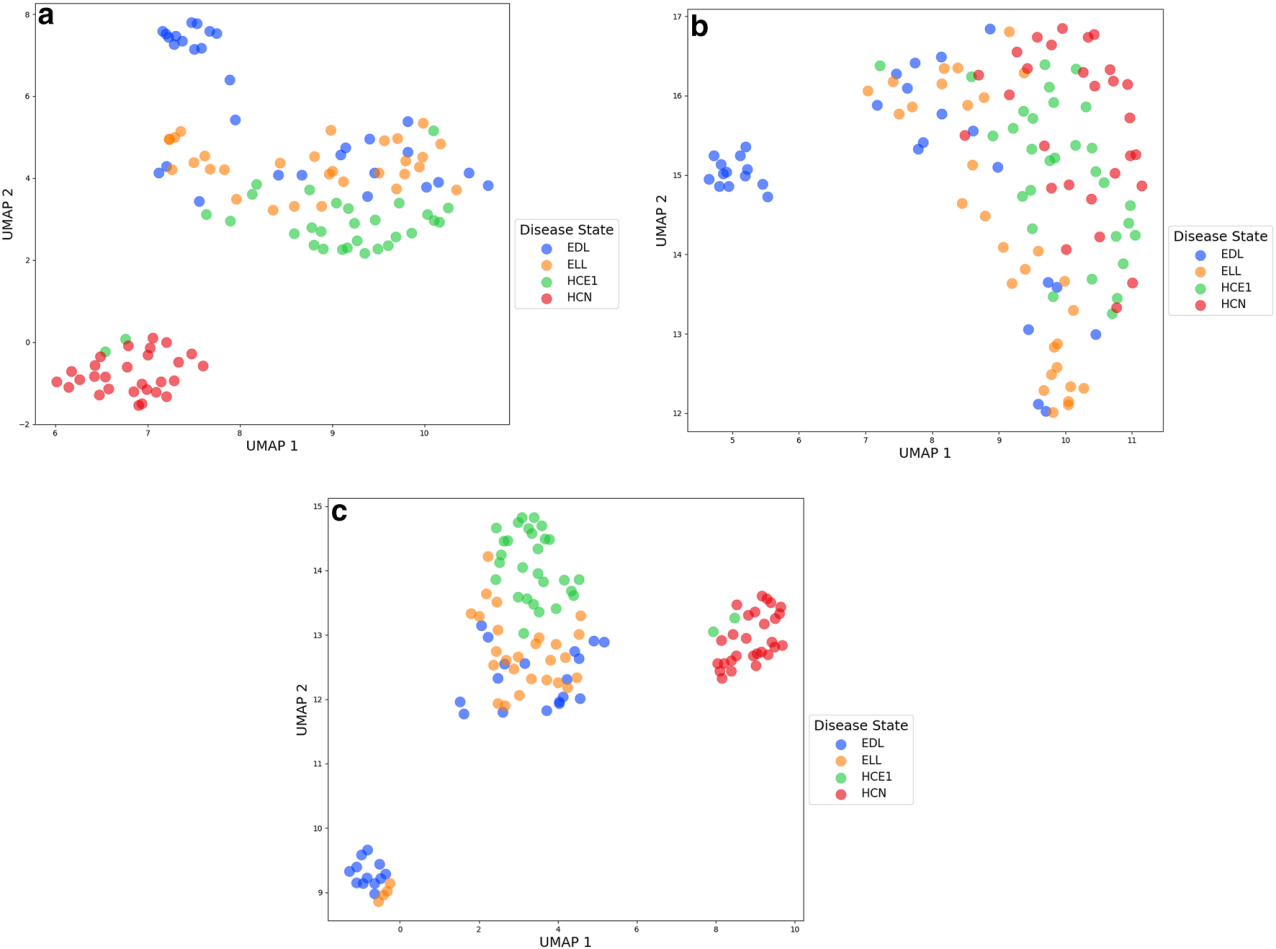
(**a**) UMAP visualization of log transformed and KNN imputed LC-MS data from training samples. *EDL* early disseminated Lyme disease, *ELL* early localized Lyme disease, *HCN* healthy control non-endemic, *HCE1* healthy control endemic site 1. (**b**) UMAP visualization of log transformed and KNN imputed LC-MS data from training samples post IFR. *EDL* early disseminated Lyme disease, *ELL* early localized Lyme disease, *HCN* healthy control non-endemic, *HCE1* healthy control endemic site 1. (**c**) UMAP visualization of log transformed and KNN imputed LC-MS data from training samples restricted to the features found by IFR. *EDL* early disseminated Lyme disease, *ELL* early localized Lyme disease, *HCN* healthy control non-endemic, *HCE1* healthy control endemic site 1.

The structure of the training samples’ data generated with the four transformation/imputation schemes was visualized by UMAP. As exemplified in Fig. 1a with data generated by log transformation and KNN imputation there was a clear separation of the early Lyme disease and the healthy control samples. However, there was a more pronounced separation of the healthy control group based on the site of sample collection. To remove those features responsible for the separation of the two healthy control groups, IFR with SSVM was applied until the mean BSR of a twofold cross-validation fell below 60% for classification of healthy control samples based on collection site. The number of features that contributed to the batch effect was dependent on the transformation/imputation method applied. Specifically, 2198, 206, 682 and 147 were identified from the log/knn, median-fold change/knn, standard/knn and raw/knn methods, respectively. Once removed from the original 4851 feature list UMAP visualization demonstrated that the batch effect disappeared for the healthy control samples (Fig. 1b). Additionally, the early disseminated Lyme disease (EDL) and early localized Lyme disease (ELL) groups remained together with a small subgroup of EDL remaining separated. Conversely, when UMAP was applied to the to the 2198 log/knn features removed by IFR a distinct separation occurs between the healthy controls based on sample collection site, but there was still separation between early Lyme disease and healthy controls. Thus, those features that were responsible for the healthy control batch effect also possessed the ability to separate samples based on disease state (Fig. 1c). Refer to Supplemental Fig. S1a–c in the Supplemental Material for UMAP visualizations of the data pre-IFR, post-IFR, and restricted to IFR features for each of the 3 other transformation/imputation methods used.

### Biomarker selection

**Figure 2:**
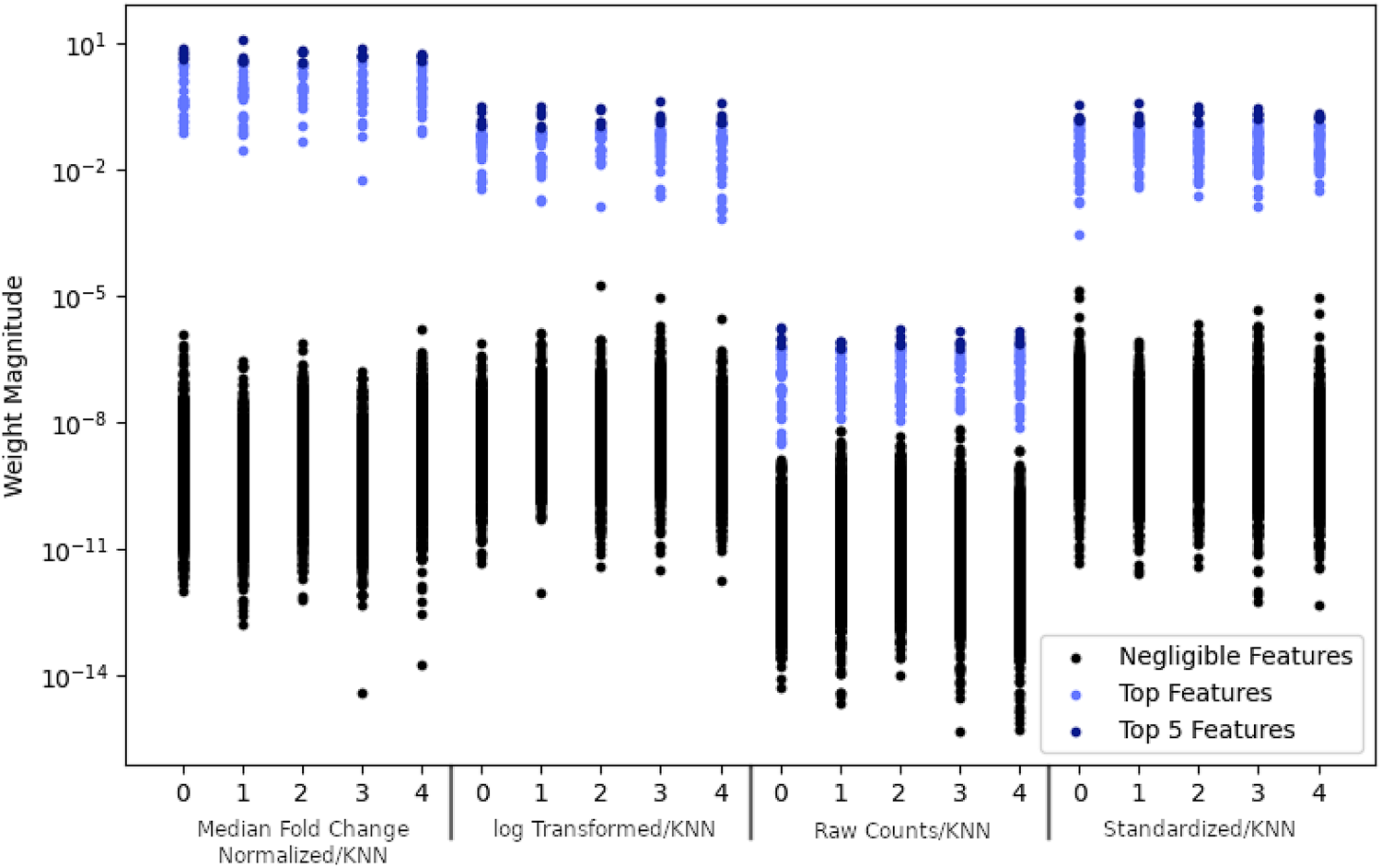
Magnitude of weights in SSVM model used in *k*FFS on training samples. The labels at the bottom indicate the transformation/imputation scheme used on the data, while the numeric ticks indicate the fold in *k*FFS.

**Figure 3:**
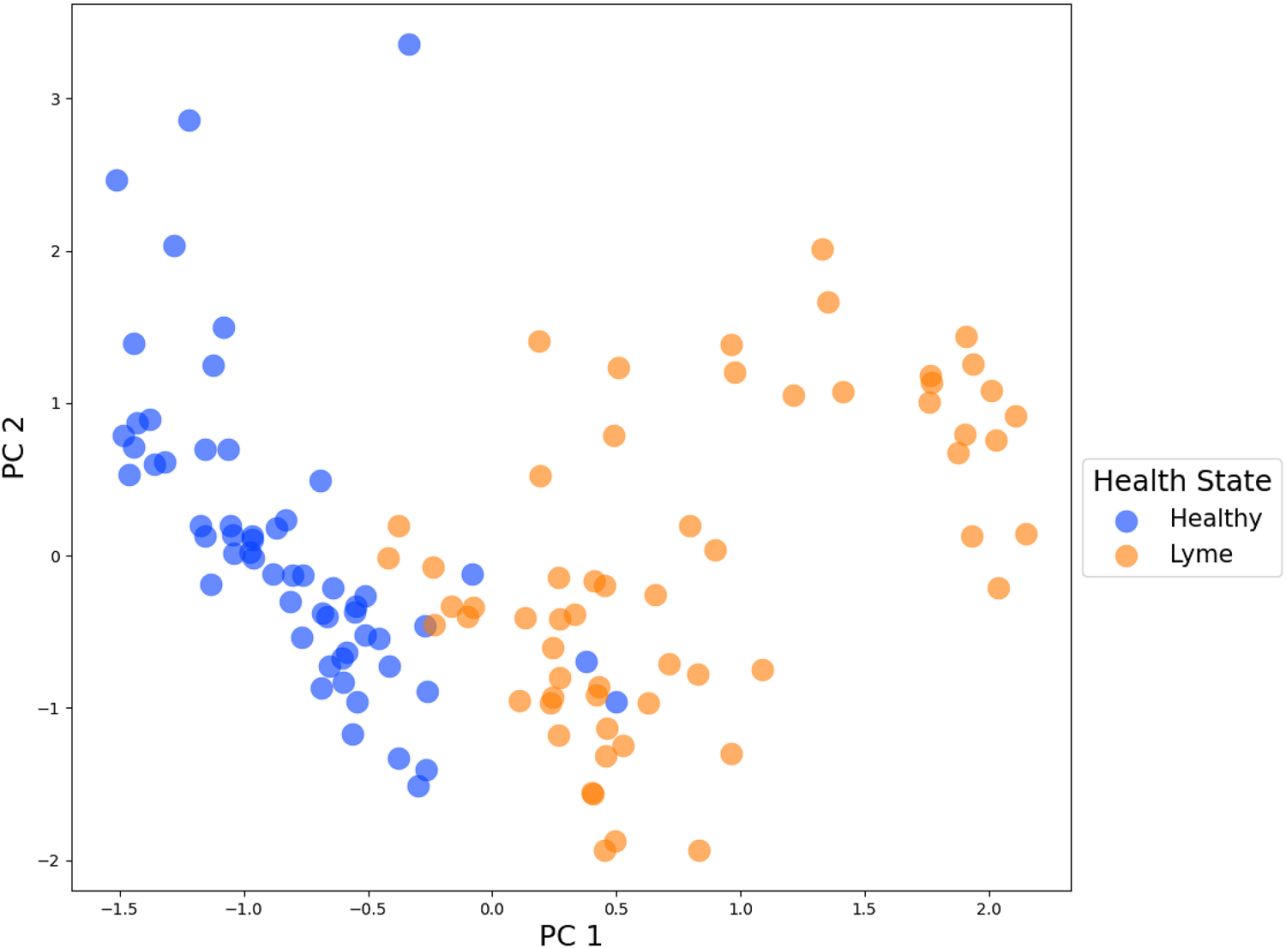
PCA visualization of log transformed and KNN imputed LC-MS data from training samples restricted to the optimal 45 features found by *k*FFS.

SSVM was applied with kFFS to select features that could populate an early Lyme disease versus healthy control biosignature. This process was performed using the features that remained after correcting the healthy control batch effect, see the “Results” section for details on the number of features removed for each method. This process was applied independently for each data set derived with the four transformation/imputation schemes. An evaluation of feature weights from each fold of SSVM revealed a clear separation between discriminatory and non-discriminatory features for all transformation/imputation schemes (Fig. 2). The smallest separation between discriminatory and non-discriminatory features occurred with the data obtained by the raw/knn scheme. Across all five SSVM folds, a total of 116, 48, 132, and 3164 features from the log/knn, median-fold change/knn, standard/knn, and raw/knn schemes, respectively, were defined as discriminatory for early Lyme disease. The accuracy of each SSVM model was assessed by fivefold cross-validation (Table 1), and revealed an accuracy of greater than 92%, regardless of the transformation/imputation scheme. The standard/knn scheme produced the highest mean accuracy (98.0%, 1.4%) with 13 top discriminatory features selected for separating early Lyme disease and healthy control groups. To limit the number of features included in a final biosignature we selected the top five discriminatory features across each SSVM fold for each transformation/imputation scheme. Once overlapping features were removed, 45 distinct biomarkers were selected (Table 2). Figure 3 validates the 45-feature biosignature on the training samples—showing a clear separation between the healthy and Lyme disease classes.

**Table 1:** Fivefold LCMS accuracy and standard deviation scores for several transformation and imputation schemes post feature selection.

Method	Mean fivefold accuracy (%)	Standard deviation (%)
Raw peak areas/KNN imputed	95.6	2.8
Standardized/KNN imputed	98.0	1.4
Log transformed/KNN imputed	97.6	1.6
Median-fold change normalized/KNN imputed	92.9	2.4

**Table 2:** Biomarkers selected by *k*FFS on training samples. The RT column indicates the retention time in seconds.

Method	RT (s)	m/z	Percent missing	Occurence	Notes
None/KNN	1103.504	481.349	0.00	5	Targeted m/z 480.3453
	1256.933*	469.389*	0.00	4	
	255.29*	227.087*	0.00	3	
	1172.216	746.563	14.41	3	
	96.231	120.081	5.93	1	Targeted m/z 166.0862
	134.919	188.069	0.00	1	Targeted m/z 205.09718
	958.025	244.263	0.00	1	
	240.743 *	247.142*	0.00	1	
	684.719	314.157	0.00	1	Targeted m/z 313.1535
	1184.953	341.248	0.00	1	
	1321.413	449.266	0.00	1	
	710.183	472.239	0.85	1	Targeted m/z 471.7369
	1165.713	508.377	0.00	1	
	845.998*	831.646*	0.00	1	
Median fold change/KNN	1018.741	174.131	0.85	4	
	255.29*	227.087*	0.00	4	
	748.564	1240.487	0.00	4	Not targeted, Isotopic peak of m/z 1238.496
	240.743*	247.142*	0.00	2	
	1256.933*	469.389*	0.00	2	
	1164.732	470.352	0.00	2	
	845.772	831.846	0.85	2	
	959.672	286.144	0.00	1	
	1195.622	331.225	0.00	1	
	891.151	829.697	0.00%	1	
	926.235	1086.303	0.00%	1	
	746.33	1238.496	2.54	1	
Log/KNN	737.416	280.151	5.08	5	
	739.352*	152.016*	0.00	4	
	1129.774	803.572	22.03	4	
	739.409	238.089	38.14	2	
	642.845	358.242	0.85	2	
	721.821*	504.337*	0.00	2	
	835.911	1042.803	7.63	2	Targeted m/z 1042.5782
	146.315	181.07	0.85	1	
	1034.796	567.402	0.85	1	Targeted m/z 566.3996
	1078.422	786.549	14.41	1	Targeted m/z 785.5421
	837.161	834.244	1.69	1	
Standard/KNN	967.457	194.117	1.69	4	
	1045.362	478.348	4.24	3	
	721.821*	504.337*	0.00	3	
	739.352*	152.016*	0.00	2	
	255.162	169.084	0.00	2	
	984.816	174.127	2.54	2	Not targeted, not present in both LCMS runs
	1231.212	429.322	2.54	2	Targeted m/z 428.3219
	758.53	671.999	5.93	2	Targeted m/z 670.9956
	1179.631	293.401	0.85	1	
	1192.645	317.407	1.69	1	Targeted m/z 317.2475
	1016.034	493.353	2.54	1	
	1711.489	814.687	0.00	1	Targeted m/z 813.6872
	954.18	1569.349	0.00	1	Not targeted, atypical MS spectra
	845.998*	831.646*	0.00	1	

The M/Z column indicates the mass divided by charge of the metabolite. The percent missing column indicates the percentage of samples that were missing the specific feature. The occurrence column indicates how many times the feature occurred across the fivefold in *k*FFS. The method indicates the normalization/imputation method used. A (*) on a feature indicates that it was picked more than once across methods.

### Lyme classification

Here we present the results of our classification experiment on test samples. The test samples were comprised of a separate set of early Lyme disease patient samples obtained from NYMC and healthy control samples obtained from NYMC and Tufts. These test samples were also analysed using LCMS at a separate time than the training samples. For this experiment both training and test LCMS samples were targeted in Skyline at the 42 biomarkers derived from the 45 biomarkers described above; see the “Methods” section for more details. After $\log$ transforming the features and training an SSVM classifier on the entire training set, we measured the classifiers performance on the set of 118 sequestered test samples.

Labeling positive as Lyme disease and negative as control, we recorded a BSR of 98.13%, a specificity (TNR) of 100.00%, and a sensitivity (TPR) of 96.25%. The confusion matrix can be viewed in Table 3, and all the related statistical test scores can be view in Table 4. Repeating our same pipeline with 42 randomly selected features, including manual inspection, we obtain a high training sensitivity and specificity (98.28%, 98.33%). For the test statistics we obtain a test specificity of 100.00%, but test sensitivity suffers greatly (36.25%)—classifying almost all the samples as healthy.

**Table 3:** Confusion matrix for classification of test samples restricted to 42 selected biomarkers with LCMS classifier using log normalized features.

		Predicted Lyme		Predicted healthy	
True Lyme	ELL	40	77	0	3
	EDL	37		3	
True healthy	HCE1	0	0	30	38
	HCE2	0		8

**Table 4:** Statistical scores (lyme = positive) for classification of test samples restricted to 42 selected biomarkers with LCMS classifier using log transformed features.

Scoring method	Score (%)
Test sensitivity (TPR)	96.25
Test specificity (TNR)	100.00
Test false discovery rate (FDR)	0.00
Test false omission rate (FOR)	7.32
Test accuracy	97.46
Test balanced success rate (BSR)	98.13

**Figure 4:**
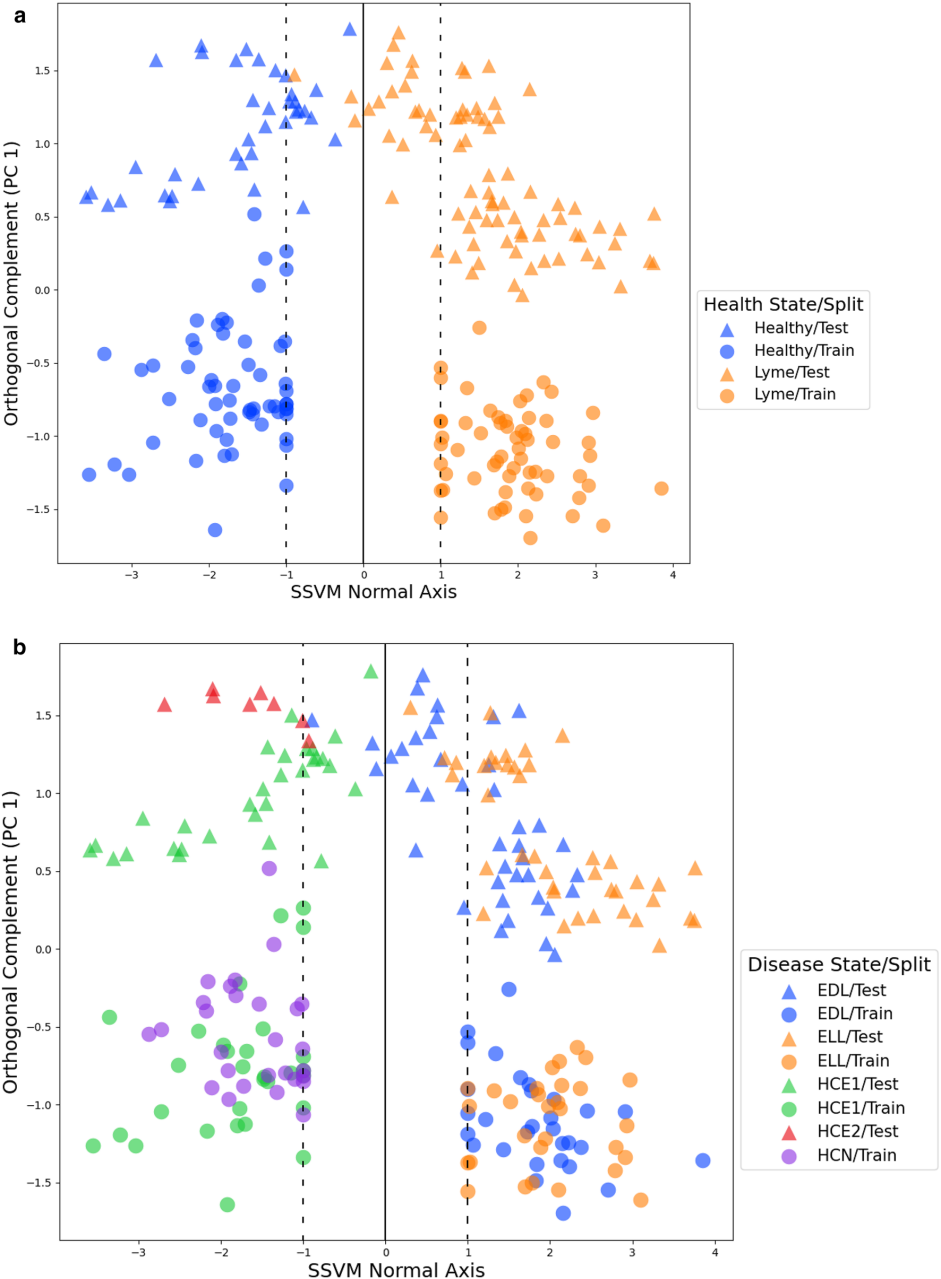
(**a**) Projection of log transformed health state labeled training and test samples onto SSVM hyperplane normal, represented as the *x*-axis. The *y*-axis represent the first principal component in the PCA decomposition of the training and test samples projected onto the orthogonal space of the hyperplane normal. The solid line indicates the hyperplane boundary, or decision boundary. Relative distance from the decision boundary indicates how strong the classification is; further is stronger, while closer is weaker. The dotted lines indicate the hyperplane margins. (**b**) Projection of log transformed disease state labeled training and test samples onto SSVM hyperplane normal, represented as the *x*-axis. The *y*-axis represent the first principal component in the PCA decomposition of the training and test samples projected onto the orthogonal space of the hyperplane normal. The solid line indicates the hyperplane boundary, or decision boundary. Relative distance from the decision boundary indicates how strong the classification is; further is stronger, while closer is weaker. The dotted lines indicate the hyperplane margins. *EDL* early disseminated Lyme disease, *ELL* early localized Lyme disease, *HCN* healthy control non-endemic, *HCE1* healthy control endemic site 1, *HCE2* healthy control endemic site 2.

Figure 4a,b show the training and test samples projected onto the hyperplane normal of the SSVM training model, along with a 1-dimensional PCA embedding of the orthogonal space to the hyperplane normal. As confirmed by Table 3, we see that all 3 Lyme disease samples misclassified as healthy were EDL. In general, we see that EDL is closer to the hyperplane (decision) boundary than its Lyme counterpart ELL; of the healthy samples, healthy control endemic site 1 (HCE1) were closest to the hyperplane (decision) boundary. When viewing the data parallel to the hyperplane of the SSVM model we noticed that there is a significant batch effect between training and test samples.

### Metabolite class validation

The biological relevance of the 42 biomarkers selected by SSVM using the training data were further investigated by LCMS/MS. Of the 42 biomarkers, MS/MS spectra could be obtained for 33 (Table 5). Using the MS/MS data biomarkers, some level of structural identification was achieved for 17 features, with eight having a level 1 or 2 structure identification^31^. These 17 features fell into the following metabolite super classes: organic acids and derivatives, organoheterocyclic compounds, alkaloids and derivatives, organic oxygen compounds, lipid and lipid-like molecules, and organic polymers.

**Table 5:** MSMS results of selected biomarkers selected by *k*FFS. The RT column indicates the retention time in minutes.

RT (m)	m/z	MS/MS	Structural ID	Level	Description
2.46	181.070201	Yes	Theobromine	1	Organoheterocyclic compounds/imidazopyrimidines/purines and purine derivatives
2.78	205.09718	Yes	Tryptophan	1	Organoheterocyclic compounds/indoles and derivatives/indolyl carboxylic acids and derivatives
16.09	286.143724	Yes	Piperine	1	Alkaloids and derivatives
1.81	166.0862	Yes	Phenylalanine	1	Organic acids and derivatives/carboxylic acids and derivatives/amino acids, peptides, and analogues
11.42	313.1535	Yes	Phe–Phe	2	Organic acids and derivatives/carboxylic acids and derivatives/amino acids, peptides, and analogues
19.92	317.247506	Yes	14(15)-Epoxy-5Z,8Z,11Z-eicosatrienoic acid [M-H2O]+	2	Lipids and lipid-like molecules/Fatty acyls/fatty acids and conjugates
19.54	508.377209	yes	PC(O-18:0/0:0)	2	Lipids and lipid-like molecules/glycerophospholipids/glycerophosphocholines
18.47	480.3453	Yes	PC(P-16:0/0:0)	2	Lipids and lipid-like molecules/glycerophospholipids/glycerophosphocholines
4.7	227.087183	Yes	Na+ adduct of lactone (similar fragmentation to cis-jasmone)	3	Organic oxygen compounds/organooxygen compounds/carbonyl compounds
2.74	247.142426	Yes	Related to tryptophan	3	Organoheterocyclic compounds/indoles and derivatives/indolyl carboxylic acids and derivatives
21.13	469.389367	Yes	Unsaturated alkyl chain	3	Lipids and lipid-like molecules/fatty acyls/fatty acids and conjugates
14.96	829.696851	Yes	Peptide	3	Organic polymers/polypeptides
14.13	831.845956	Yes	Peptide	3	Organic polymers/polypeptides
15.46	1086.303121	Yes	Peptide	3	Organic polymers/polypeptides
12.55	1238.496491	Yes	Peptide	3	Organic polymers/polypeptides
19.63	746.563218	Yes	Peptide	3	Organic polymers/polypeptides
14.13	831.646014	Yes	Peptide	3	Organic polymers/polypeptides
12.42	152.016163	Yes		4	
4.27	169.084118	Yes		4	
16.99	174.130592	Yes		4	
12.42	238.089239	Yes		4	
19.7	293.400601	Yes		4	
19.58	341.248414	Yes		4	
10.78	358.242021	Yes		4	
20.6	428.3219	Yes		4	
11.79	471.7369	Yes		4	
17.63	478.347583	Yes		4	
16.98	493.352828	Yes		4	
12.12	504.336795	Yes		4	
17.3	566.3996	Yes		4	
12.64	670.9956	Yes		4	
18.04	785.5421	Yes		4	
16.23	194.117098	No			
16.03	244.263279	No			
12.4	280.151108	No			
19.92	331.224627	No			
22.23	449.266367	No			
19.53	470.351806	No			
18.95	803.571864	No			
28.49	813.6872	No			
14.13	834.244267	No			
13.84	1042.5782	No			

The M/Z column indicates the mass divided by charge of the metabolite.

Manual inspection of the 45 biomarkers selected by SSVM revealed that monoisotopic peaks were not selected in the original list and thus the monoisotopic m/z values were used to replace the original m/z values as indicated in Table 2. Upon evaluation of MS/MS spectra for feature ID 902 (m/z 317.4072, RT 19.92 min), it was discovered that a co-eluting ion with m/z 317.2475 had a higher abundance and matched the spectra of the [M+H-H2O]+ adduct of 14(15)-Epoxy-5Z,8Z,11Z-eicosatrienoic acid in the NIST database. This m/z was present in the list of discriminatory features identified using *k*FFS, but was not in the cut-off used to select the top 42 features. Thus, the m/z 317.4072 ion was replaced by m/z 317.2475 as a discriminatory feature.

Additionally, there were three biomarkers that were removed from Table 2 following manual inspection. Specifically, feature ID 4698 (m/z 1240.487, RT 748.564 s) was the isotopic peak for another feature already included; feature ID 4694 (m/z 1238.496, RT 746.33 s). Feature ID 269 (m/z 174.127, RT 984.816 s) was not present in both training and test LCMS runs. Feature ID 4846 (m/z 1569.349, RT 954.18 s) had atypical MS spectra. The remaining 42 features were present among all training and test samples.

## Discussion

Our end-to-end pipeline starts with a large set of features detected through a non-targeted metabolomics experiment and produces an optimal set of targeted discriminatory features capable of identifying out-of-sample Lyme disease patients with high accuracy. This pipeline has significant potential for the development of additional ML based LCMS diagnostic tests.

In particular, our SSVM classification model classified a sequestered batch of LCMS samples as healthy or having Lyme disease with a 98.13% balanced success rate, 96.25% sensitivity, and 100.00% specificity, see Table 4. The high classification results are strengthened by the apparent batch effect between training and test samples post-Skyline targeting, see Fig. 4a,b. This indicates that our features from the training generalize and that we may be able to classify incoming samples from different batches with high accuracy.

**Figure 5:**
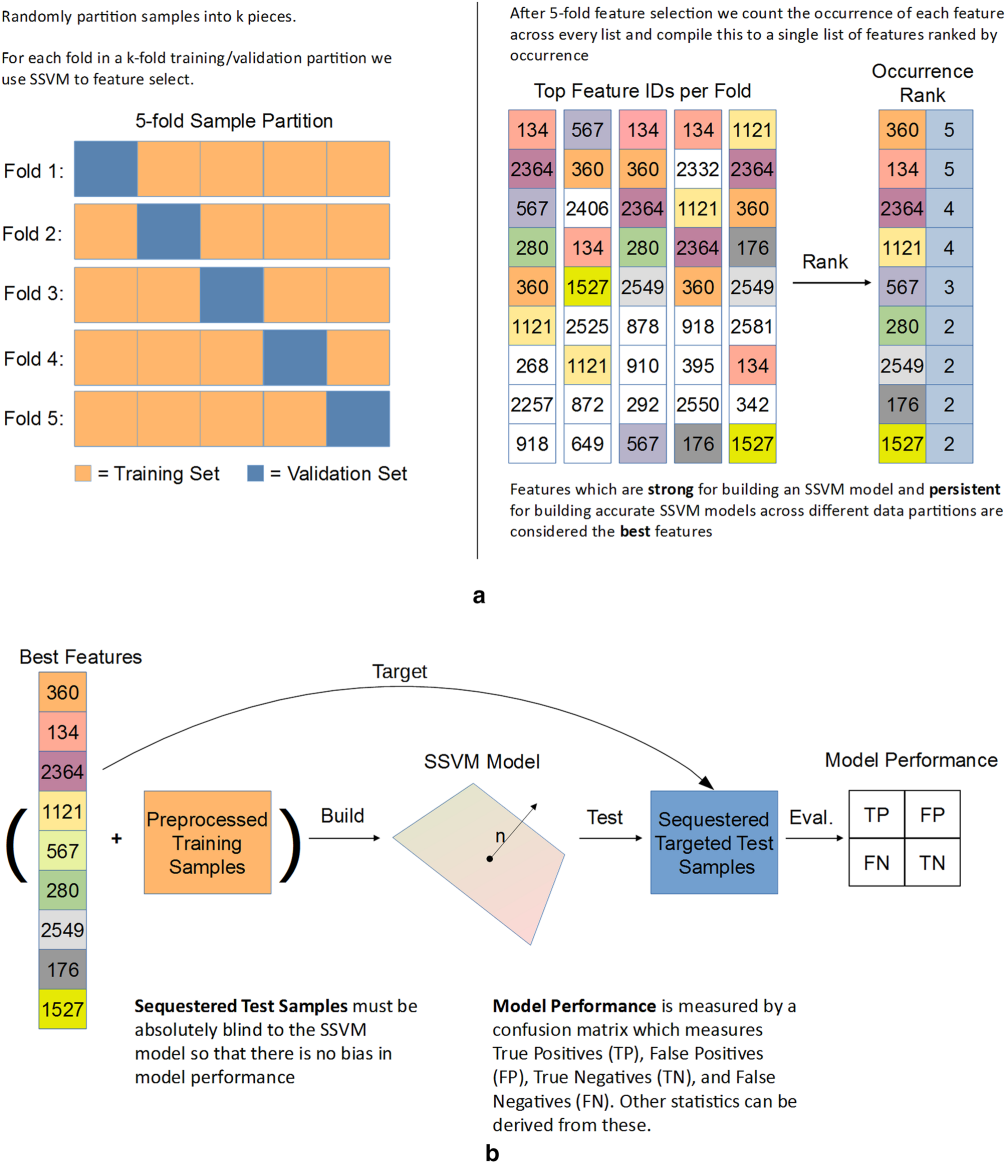
(**a**) Diagram of *k*FFS. (**b**) Diagram of building the final model.

Relative to the 44 LC-MS biomarkers discovered and LASSO diagnostic developed in Molins et al. our SSVM diagnostic shows an 8.35% increase in test sensitivity and a 5.00% increase in test specificity^5^. Our results are strengthened by the fact that in Molins et. al. all of the test data participated in the same LC-MS runs as the training data—which was used to build their final LASSO model. Our test samples were completely sequestered from the training data which includes the step of being processed by LC-MS (3 month gap), so that none of test samples were run with any of the training samples. Our diagnostic greatly outperforms the models developed in Clarke et al. on 50 peripheral blood mononuclear cell (PBMC) RNA seq biomarkers^32^. The highest scoring logistic regression model on 50 biomarkers of Clarke et al. yielded approximately (50% TPR, 0% FPR) and (100% Precision, 50% Recall) as observed from their ROC and Precision-Recall curves; this is in contrast to our models (96.25% TPR, 0.00% FPR) and (100.00% Precision, 96.25% Recall). Clarke et al. has each of their batches represented in both training and test—yielding a significantly weaker model than ours, where training and test data are split into separate LCMS batches.

Pegalajar et al. tests the diagnostic capability of their positive-ion and negative-ion mode LC-MS urine biosignatures for discriminating EDL and Healthy controls using linear discriminant analysis (LDA) in a leave-one-out (LOO) experiment where training data and test data are run in the same batch^11^. Our SSVM diagnostic outperforms their best results of 86% TPR and 86% TNR using their positive-ion mode biosignature ($\le$ 1262 metabolites). Huang et al. performs analogous experiment to ours, with weaker performance, to discover a metabolic biosignature which can discriminate between early-stage lung adenocarcinoma (LA) and healthy controls^33^. For their sparse classification model they used an elastic net regularized logistic regression model consisting of a 7 metabolite biosignature—recording 88.57% sensitivity and 91.30% specificity on a sequestered batch of test samples.

Not only did our 42 features classify Lyme disease patients with high accuracy, our features were present in all samples upon manual inspection and they are tied to metabolic processes altered during Lyme disease. The included features belong to glycerophospholipid, eicosanoid, tryptophan and phenylalanine metabolic pathways previously shown to be altered during Lyme disease^10,11,24^. As these pathways have come up multiple times, further investigation into the classification efficacy of all metabolites in these pathways may provide a more robust classifier for Lyme disease. Our null experiment, see the “Results” section, shows that our features generalize to a separate batch of samples by maintaining consistency between training and test statistics. In the case of the random features, the SSVM model over-fits to the data and is unable to capture the actual signal of the disease state with respect to those features. Additional analyses of how these metabolites classify Lyme disease patients from clinical controls with symptoms, but not Lyme disease are required to understand the real diagnostic potential of these features.

More data needs to be acquired and further analysis needs to be performed to assess the efficacy of the classification model on health states outside of Lyme disease. For example, the model we built used only healthy controls, but it would valuable to see the classification results of patients infected with the common cold or influenza. In future work we propose to extend this test beyond distinguishing between suspected Lyme disease and actual Lyme disease to more specific disease identification.

## Methods

### LCMS analysis

Serum sample LCMS data acquired previously was utilized^24^. Detailed methods for metabolite extraction and LCMS analysis can be found in the cited publication.

### Data partitioning

Early Lyme disease and healthy control serum samples, previously analyzed by LCMS as two separate batches, were utilized in this study^24^. These two independently processed batches formed our 118 training samples and 118 sequestered test samples respectively. Samples were categorized by the health state labels: EDL, ELL, healthy control non-endemic (HCN), HCE1, and healthy control endemic site 2 (HCE2). Training samples were partitioned as 30 EDL, 30 ELL, 28 HCN, and 30 HCE1. Test samples were partitioned as 40 EDL, 40 ELL, 30 HCE1, and 8 HCE2. We label a sample as Lyme disease if it belongs to either the ELL or EDL group, and label a sample as healthy if it belongs to the HCE1, HCN, or HCE2 group.

### Untargeted and targeted peak identification

For untargeted feature selection, raw data files were converted into mzML format files using MSConvert (Proteowizard) and then processed using XCMS (3.6.2) in R (3.6.1)^25,34^. Peak detection was performed using the centWave algorithm^35^. Default parameters were used except for ppm = 30, peakwidth = c(10,30), and noise = 2000). Peak alignment by retention time was carried out using the obiwarp method with binSize = 0.6 and specifying the centerSample as the sample that was measured in middle of the LCMS run^36^. Quality control included manual inspection of plots of total ion counts and specified peaks by retention time. Peaks were grouped using the peak density method with default parameters except bw = 5 and minfrac = 0.4^25^.

Features selected by *k*FFS were manually inspected to determine peak quality, whether the monoisotopic peak was chosen, any possible adducts, and feature presence in both runs. After manual evaluation, good quality features were targeted in both the training and test sets using Skyline with suggested settings^28,37^. Each peak was manually evaluated to ensure correct integration before exporting peak area values.

### Cleaning, imputing, and normalizing

As a first step, any metabolites which were missing in more than 80% of samples across each class of healthy or Lyme disease were removed. No features in our list met this criterion and so no features were removed. All samples with missing values were imputed by the KNN algorithm^38^. KNN imputes missing data in a sample by finding its *k*-nearest neighbors, taking the mean of a feature with respect to its neighbors, and then imputing that value for the missing feature. Wahl et al. concludes that KNN imputation performs well across several evaluation schemes and computationally takes less resources^39^. Modified versions of the KNN imputation algorithm, such as normalized No-Skip KNN (NS-KNN), have been proposed and may even outperform the standard algorithm for real datasets when a significant portion of the missing data is Missing Not at Random (MNAR) type^38^. For this particular application we used $k=5$ and implemented the algorithm via the python package missingpy.

Once imputed, the samples were transformed by either the $\log _2$ transform, standardization, median-fold change normalization, or using raw peak areas^40^. Standardization is defined as shifting and scaling each feature so that its mean is 0 and its variance is 1 across samples. These transformation schemes were chosen to be the best with respect to the classification accuracy of the SSVM model on the training data, amongst other transformation schemes such as quantile normalization^26^; see the Supplemental_Data directory in our github repository for our complete transformation/imputation experiment^29^.

### Sparse support vector machines

We classify samples into two classes of healthy, $C_-$, and Lyme disease, $C_+$, using a variation of SVM called SSVM^8,41^. Each sample $\mathbf {x}$ can be viewed as vector living in $\mathbb {R}^n$ where *n* is the number of features/biomarkers/measurements. SVM classifies samples by first constructing a hyperplane $\mathbf {H}\subset \mathbb {R}^n$ which best separates the training samples into $C_-$ and $C_+$. SSVM alters SVM by finding a hyperplane which, in addition to separating the two classes, uses relatively few features compared to the entire feature space. Explicitly, we solve the convex optimization problem

1 $$\begin{aligned} \min _{\mathbf {w},\varvec{\xi },b} \left\Vert \mathbf {w}\right\Vert _1 + C\varvec{e}^T\varvec{\xi }\quad \text {subject to}\quad \mathbf {Y}\left( \mathbf {X}\mathbf {w}-b\mathbf {e}\right) +\varvec{\xi } \ge \mathbf {e},\,\, \varvec{\xi }\ge \mathbf {0}, \end{aligned}$$

where $\mathbf {X}$ is the $m\times n$ matrix whose *i*th row $\mathbf {X}^{(i)}\in \mathbb {R}^n$ is the feature vector for the *i*th sample, $\mathbf {Y}$ is the $m\times m$ diagonal matrix whose entries are either $+\,1$ or $-\,1$ corresponding the class labels of samples, $\varvec{\xi }\in \mathbb {R}^m$ is the vector of penalties for samples violating the hyperplane boundary, *C* is a tuning parameter for balancing the misclassification rate against the sparsity, $\mathbf {e}$ is the vector of all 1’s in the appropriate dimension space, $\mathbf {w}$ is the normal vector to the hyperplane $\mathbf {H}$, and *b* is the scalar affine shift of the hyperplane $\mathbf {H}$. It is known that minimizing the 1-norm of $\mathbf {w}$ promotes sparsity in the components of $\mathbf {w}$^42,43^. That is $\mathbf {w}$ will have relatively few large components while its many other components will be near zero, see Fig. 2. It appears to be a special feature of SSVM that there is an abrupt drop in feature size, i.e., often on the order of a 100–1000 factor reduction, see Fig. 2. Features corresponding to large components in $\mathbf {w}$ are chosen to build a sparse model. We solve (1) by first transforming the convex optimization problem into a linear program via a simple substitution and then applying a primal-dual interior point method using our own in-house python package calcom—provided in our github repository^29,44,45^.

### *k*-fold feature selection (*k*FFS)

We selected features/biomarkers using a new method: *k*FFS. First, we randomly partitioned training samples into *k* non-overlapping and equally-sized parts. We then chose $k-1$ parts as a training set for an SSVM classifier and then validated the classifier on the withheld part. There are *k* ways to choose $k-1$ parts from *k* parts—therefore we obtained a *k*-fold experiment, known as *k*-fold cross validation (cross-validation). For each fold of the experiment we extracted features, ordered them by the absolute value of their weight in the SSVM model, grabbed the top $p\le n$ features from each fold, collected them into a common list of features, and then ordered the list by feature occurrence across the *k* folds, see Fig. 5a. For the results of our paper we used $k=5$ and an $p=5$. Using multiple folds for feature selection brings in features from sub-populations of the data that may not be captured by using the training set as a whole. Ordering by frequency shows which of those features generalize to the entire training set.

**Figure 6:**
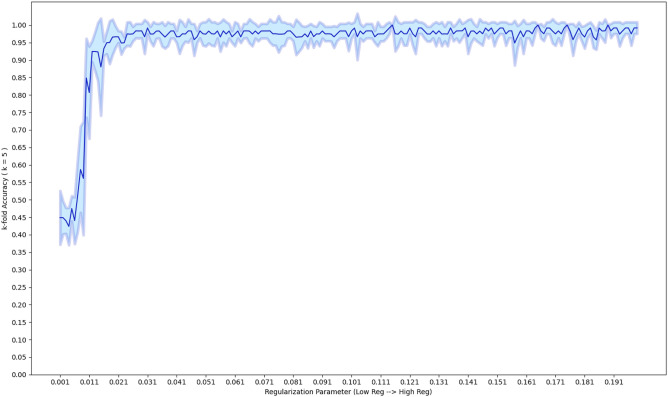
Fivefold classification accuracy of SSVM model for different values the hyper-parameter *C*. The solid line indicates the mean accuracy across fivefold while the shaded regions indicate 1 standard deviation of the accuracy.

### Batch correction

For batch correction we used an IFR technique, which we simply call IFR, to remove features discriminating between HCN and HCE1 control groups in the training set^23^. Specifically, we perform *k*FFS ($k=2$, $n=5$) between the training HCE1 and HCN groups, obtain a set of discriminatory features, remove these features, and then repeat the process until the mean 2-fold cross-validation accuracy of the SSVM classifier goes below $60\%$, see Algorithm 1.

To evaluate the efficacy of IFR for batch correction we utilized the visualization tool UMAP. UMAP attempts to embed data into a lower dimensional space so that it is approximately uniformly distributed and its local geometry is preserved^27^. UMAP does so by representing each *k*-neighborhood of a sample as a weighted graph, “gluing” these graphs together over all samples, and then approximating the resulting global structure in a lower dimensional space.

If it happens that a point has most of its neighbors from the same class or batch then this point will be pulled in that direction in the embedding; making it a great tool for visualizing batch effects in data. We used the python package umap-learn with parameters min_dist$=.1$, n_neighbors$=15$, n_components$=2$ for our UMAP visualizations. See Tran et al. for UMAP applied to several genomics data sets^46^.

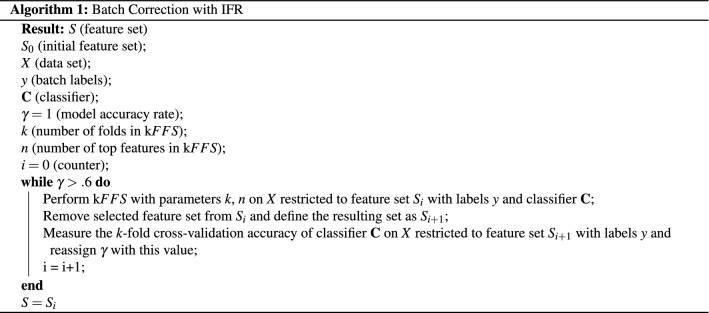



### Classification

Once we removed features for batch effects we restricted the training data to the remaining features, and we then either $\log _2$ transformed, standardized, median-fold change normalized, or did not transform the training data. Once transformed we imputed the training samples using the KNN algorithm. We performed a fivefold cross-validation experiment with an SSVM classifier, while varying the hyper-parameter *C* in Eq. (1). *C* was chosen so that it was as small as possible (promoting sparsity), while simultaneous yielding high accuracy and small variance, see Fig. 6.

We classified test samples by first restricting both the training data and test data to the selected features; found by the methods above. We restricted the samples by first targeting these features in Skyline. Once these new feature sets were obtained they were $\log _2$ transformed and a SSVM classifier was trained and tuned on all of the training samples. We then evaluated the performance of the classifier on the sequestered test samples via confusion matrix, see Fig. 5b for a diagram of the classification pipeline.

### Metabolite class validation

Confirmation of the chemical structure of selected molecular features (MF) was performed by LCMS/MS. MS/MS spectra were manually evaluated using MassHunter Qualitative software (Agilent Technologies)^47^. MS/MS spectra were compared with available spectra in Metlin and NIST databases. The level of structural identification followed refined Metabolomics Standards Initiative guidelines proposed by Schymanski et al.^31^.

## Supplementary Information

Supplementary Figures.
