# Supplementary material for: Biomarker selection and a prospective metabolite-based machine learning diagnostic for lyme disease
Source: Sci Rep. 2022 Jan 27;12:1478. doi: 10.1038/s41598-022-05451-0 (PMC8795431; doi:10.1038/s41598-022-05451-0)
Supplement: Supplementary file 1 — Supplementary Figures. [file 41598_2022_5451_MOESM1_ESM.pdf]

Supplemental Material: Biomarker Selection and a Prospective Metabolite-based Machine Learning Diagnostic for Lyme Disease

Eric R. Kehoe[2,*], Bryna L. Fitzgerald[3], Barbara Graham[3], M. Nurul Islam[3], Kartikay Sharma[1], Gary P. Wormser[4], John T. Belisle[3], and Michael J. Kirby[1,2]

[1]Department of Computer Science, Colorado State University, Fort Collins, CO 80523
[2]Department of Mathematics, Colorado State University, Fort Collins, CO 80523
[3]Department of Microbiology, Immunology & Pathology, Colorado State University, Fort Collins, CO 80523
[4]Department of Medicine, New York Medical College, Valhalla, NY 10595
[*]Eric.Kehoe@colostate.edu

ABSTRACT

We provide a pipeline for data preprocessing, biomarker selection, and classification of liquid chromatography–mass spectrometry (LCMS)) serum samples to generate a prospective diagnostic test for Lyme disease. We utilize tools of machine learning (ML), e.g. sparse support vector machines (SSVM), iterative feature removal (IFR), and k-fold feature ranking to select several biomarkers and build a discriminant model for Lyme disease. We report a 98.13% test balanced success rate (BSR) of our model based on a sequestered test set of LCMS serum samples. The methodology employed is general and can be readily adapted to other LCMS, or metabolomics, data sets.

Supplementary Figures and Tables

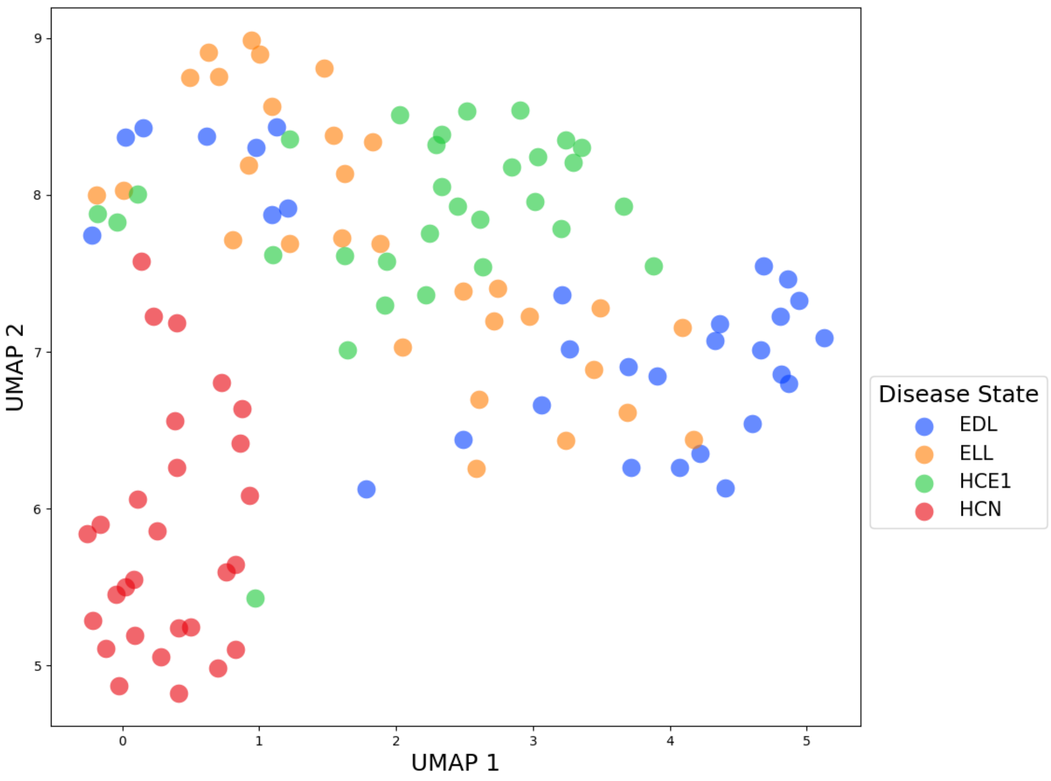

Figure S1a. UMAP visualization of standardized (mean = 0, variance = 1) and KNN imputed LC-MS data from training samples. EDL - early disseminated Lyme disease, ELL - early localized Lyme disease, HCN - healthy control non-endemic, and HCE1 - healthy control endemic site 1

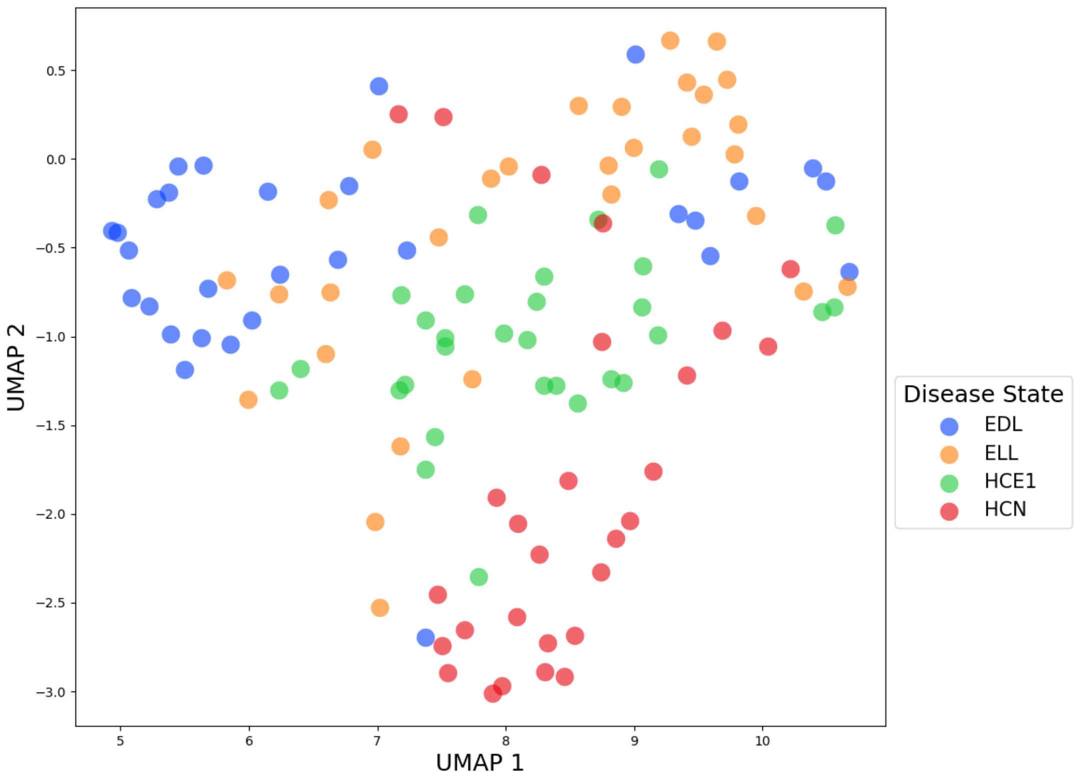

Figure S1b. UMAP visualization of standardized (mean = 0, variance = 1) and KNN imputed LC-MS data from training samples post IFR. EDL - early disseminated Lyme disease, ELL - early localized Lyme disease, HCN - healthy control non-endemic, and HCE1 - healthy control endemic site 1

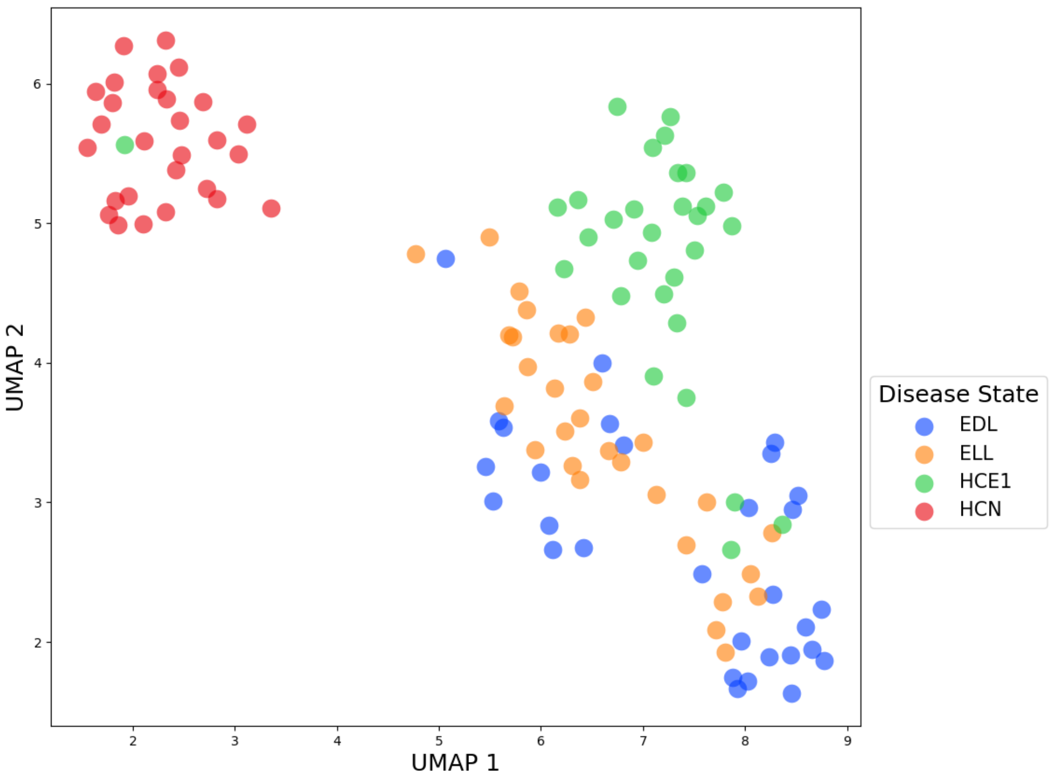

Figure S1c. UMAP visualization of standardized (mean = 0, variance = 1) and KNN imputed LC-MS data from training samples restricted to the features found by IFR. EDL - early disseminated Lyme disease, ELL - early localized Lyme disease, HCN - healthy control non-endemic, and HCE1 - healthy control endemic site 1.

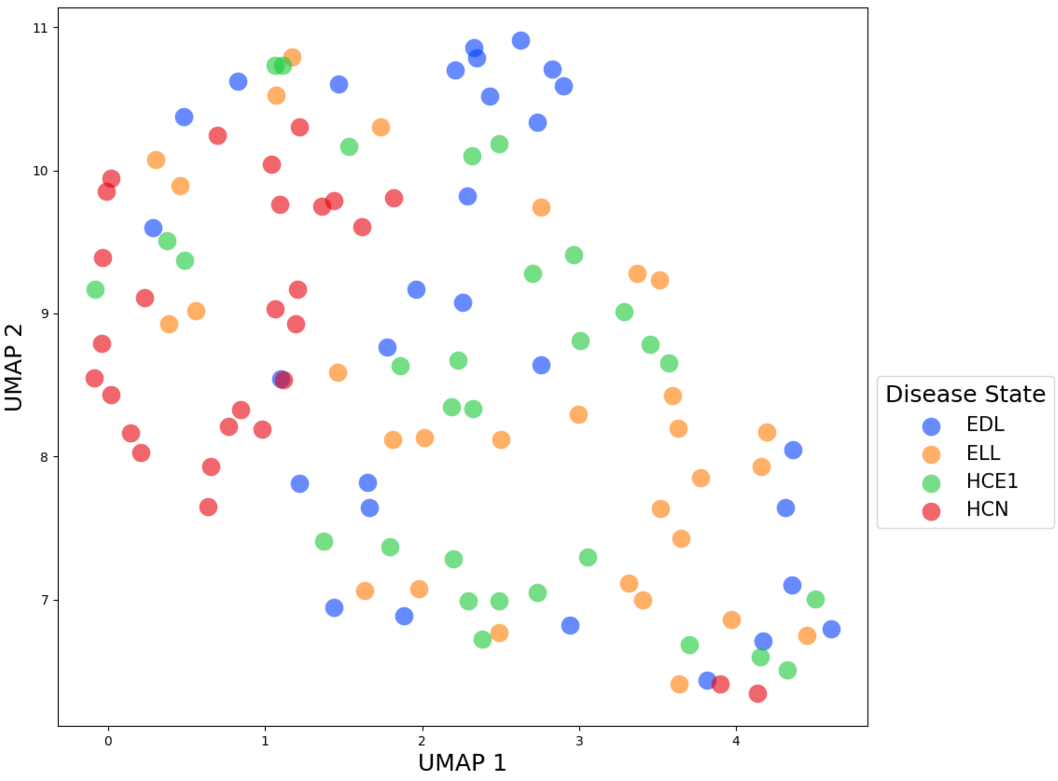

Figure S2a. UMAP visualization of median-fold change normalized and KNN imputed LC-MS data from training samples. EDL - early disseminated Lyme disease, ELL - early localized Lyme disease, HCN - healthy control non-endemic, and HCE1 - healthy control endemic site 1

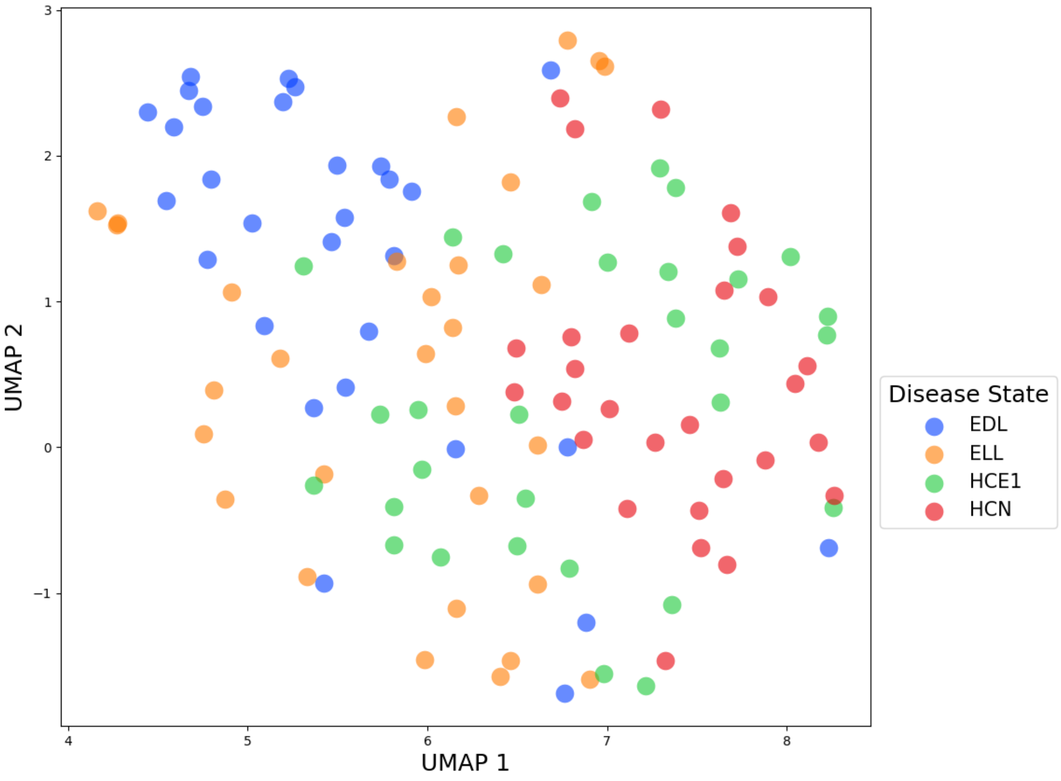

Figure S2b. UMAP visualization of median-fold change normalized and KNN imputed LC-MS data from training samples post IFR. EDL - early disseminated Lyme disease, ELL - early localized Lyme disease, HCN - healthy control non-endemic, and HCE1 - healthy control endemic site 1

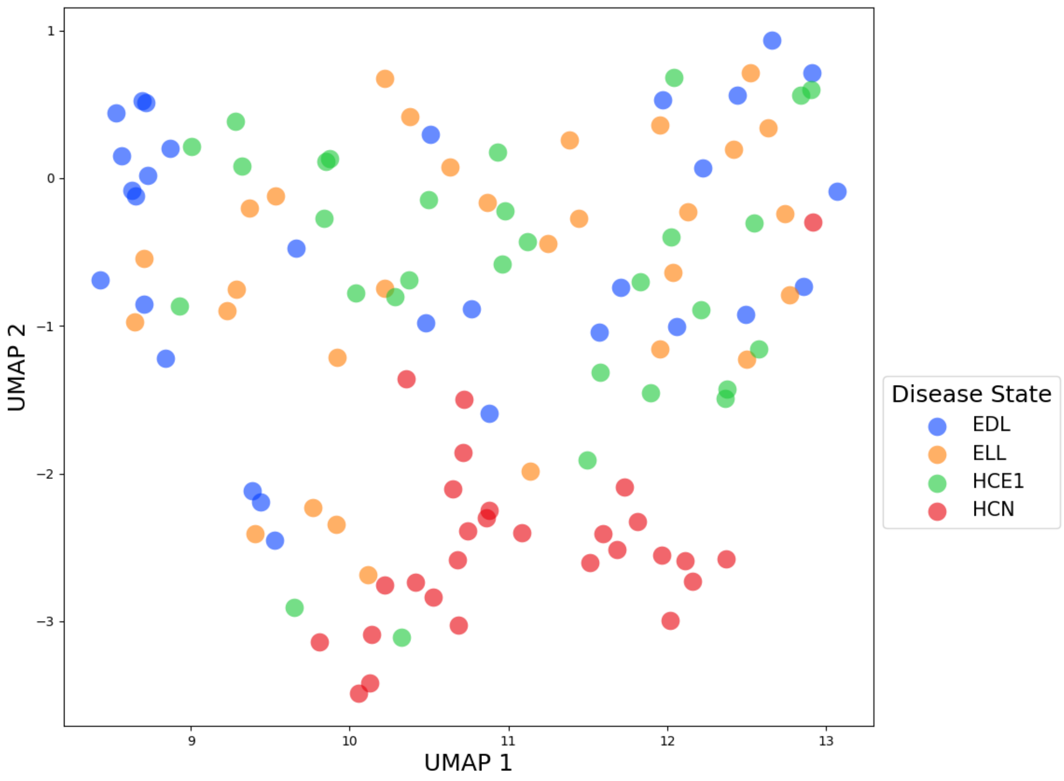

Figure S2c. UMAP visualization of median-fold change normalized and KNN imputed LC-MS data from training samples restricted to the features found by IFR. EDL - early disseminated Lyme disease, ELL - early localized Lyme disease, HCN - healthy control non-endemic, and HCE1 - healthy control endemic site 1.

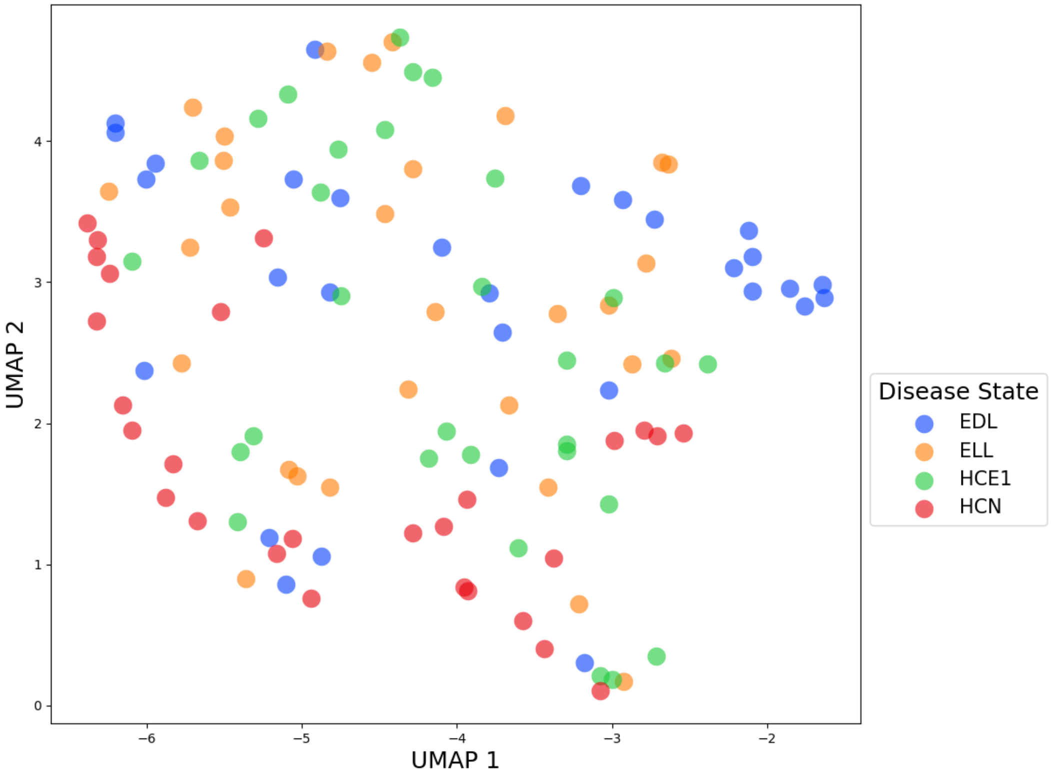

Figure S3a. UMAP visualization of KNN imputed LC-MS data from training samples. EDL - early disseminated Lyme disease, ELL - early localized Lyme disease, HCN - healthy control non-endemic, and HCE1 - healthy control endemic site 1

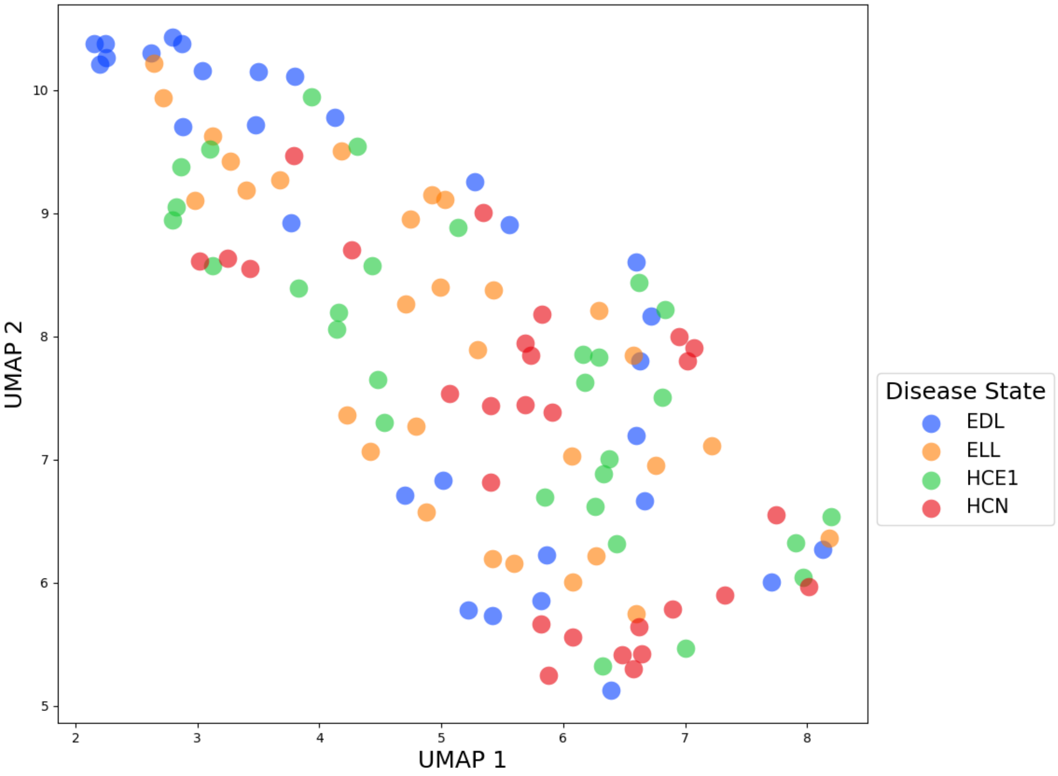

Figure S3b. UMAP visualization of KNN imputed LC-MS data from training samples post IFR. EDL - early disseminated Lyme disease, ELL - early localized Lyme disease, HCN - healthy control non-endemic, and HCE1 - healthy control endemic site 1

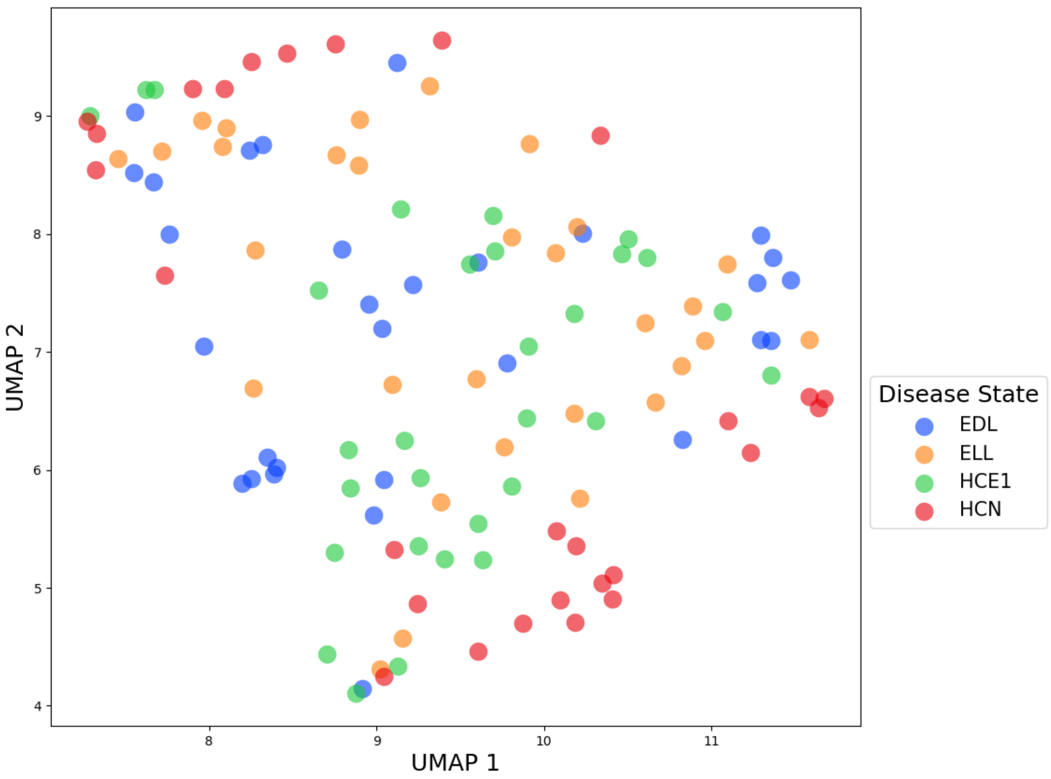

Figure S3c. UMAP visualization of KNN imputed LC-MS data from training samples restricted to the features found by IFR. EDL - early disseminated Lyme disease, ELL - early localized Lyme disease, HCN - healthy control non-endemic, and HCE1 - healthy control endemic site 1.
